# Supplementary material for: Plant traits and environment: floating leaf blade production and turnover of waterlilies
Source: PeerJ. 2017 Apr 27;5:e3212. doi: 10.7717/peerj.3212 (PMC5410161; doi:10.7717/peerj.3212)
Supplement: Data S1 [file peerj-05-3212-s001.pdf]

Raw data 1977: Plot, Date, Leaf number, Leaf length

Plot: I = HW, Nuphar lutea; J = OW, Nuphar lutea; K = OW, Nymphaea alba; L = HW, Nymphaea

Plot	Date	Leaf number	Leaf length (cm)
I	17-mei-77	1	27,5
I	24-mei-77	1	27,5
I	17-mei-77	2	30
I	24-mei-77	2	30
I	17-mei-77	3	29,5
I	24-mei-77	3	29,5
I	17-mei-77	4	29
I	24-mei-77	4	29
I	17-mei-77	5	25
I	24-mei-77	5	26,5
I	31-mei-77	5	26,5
I	7-jun-77	5	27
I	14-jun-77	5	27
I	21-jun-77	5	27
I	17-mei-77	6	28,5
I	24-mei-77	6	30
I	31-mei-77	6	30
I	7-jun-77	6	30
I	14-jun-77	6	30
I	21-jun-77	6	30
I	17-mei-77	7	27,5
I	24-mei-77	7	29
I	31-mei-77	7	29
I	7-jun-77	7	29
I	14-jun-77	7	29
I	21-jun-77	7	29
I	5-jul-77	7	29
I	24-mei-77	8	28
I	31-mei-77	8	28
I	7-jun-77	8	28
I	14-jun-77	8	28
I	21-jun-77	8	28
I	24-mei-77	9	22
I	31-mei-77	9	23,5
I	7-jun-77	9	24
I	14-jun-77	9	24
I	24-mei-77	10	30
I	31-mei-77	10	30
I	7-jun-77	10	30
I	14-jun-77	10	30
I	21-jun-77	10	30
I	31-mei-77	11	27
I	7-jun-77	11	27
I	14-jun-77	11	27
I	21-jun-77	11	27
I	31-mei-77	12	26
I	7-jun-77	12	26,5
I	14-jun-77	12	26,5
I	21-jun-77	12	28
I	5-jul-77	12	28

I	12-jul-77	12	28
I	31-mei-77	13	27
I	7-jun-77	13	29
I	14-jun-77	13	30
I	21-jun-77	13	30,5
I	5-jul-77	13	30,5
I	7-jun-77	14	30
I	14-jun-77	14	31
I	21-jun-77	14	32
I	5-jul-77	14	32
I	12-jul-77	14	32
I	19-jul-77	14	32
I	7-jun-77	15	29
I	14-jun-77	15	30
I	21-jun-77	15	31
I	5-jul-77	15	31
I	12-jul-77	15	31
I	21-jun-77	16	32
I	5-jul-77	16	32
I	21-jun-77	17	31,5
I	5-jul-77	17	31,5
I	21-jun-77	18	29
I	5-jul-77	18	31,5
I	12-jul-77	18	32
I	19-jul-77	18	32
I	26-jul-77	18	32
I	2-aug-77	18	32
I	21-jun-77	19	35,5
I	5-jul-77	19	35,5
I	21-jun-77	20	30
I	5-jul-77	20	30
I	21-jun-77	21	30,5
I	5-jul-77	21	30,5
I	21-jun-77	22	31
I	5-jul-77	22	32
I	12-jul-77	22	32,5
I	19-jul-77	22	32,5
I	26-jul-77	22	32,5
I	21-jun-77	23	29
I	5-jul-77	23	32
I	12-jul-77	23	33
I	19-jul-77	23	33
I	26-jul-77	23	33
I	2-aug-77	23	33,5
I	9-aug-77	23	33,5
I	21-jun-77	24	25,5
I	5-jul-77	24	25,5
I	12-jul-77	24	25,5
I	19-jul-77	24	25,5
I	5-jul-77	25	30,5
I	12-jul-77	25	32
I	19-jul-77	25	33
I	26-jul-77	25	33
I	2-aug-77	25	33
I	9-aug-77	25	33
I	16-aug-77	25	33

l	**5-jul-77**	26	33
l	**12-jul-77**	26	33,5
l	**19-jul-77**	26	33,5
l	**26-jul-77**	26	34
l	**2-aug-77**	26	34
l	**5-jul-77**	27	31,5
l	**12-jul-77**	27	32
l	**19-jul-77**	27	32,5
l	**26-jul-77**	27	32,5
l	**2-aug-77**	27	33
l	**9-aug-77**	27	33
l	**16-aug-77**	27	33
l	**5-jul-77**	28	30
l	**12-jul-77**	28	30
l	**19-jul-77**	28	30
l	**26-jul-77**	28	30
l	**5-jul-77**	29	30
l	**12-jul-77**	29	30
l	**19-jul-77**	29	30,5
l	**26-jul-77**	29	30,5
l	**2-aug-77**	29	30,5
l	**5-jul-77**	30	33
l	**12-jul-77**	30	34
l	**19-jul-77**	30	34
l	**26-jul-77**	30	34
l	**2-aug-77**	30	34
l	**9-aug-77**	30	34
l	**5-jul-77**	31	32
l	**12-jul-77**	31	32
l	**19-jul-77**	31	33
l	**26-jul-77**	31	33
l	**2-aug-77**	31	33
l	**12-jul-77**	32	30,5
l	**19-jul-77**	32	32
l	**26-jul-77**	32	33
l	**2-aug-77**	32	33,5
l	**9-aug-77**	32	33,5
l	**16-aug-77**	32	33,5
l	**25-aug-77**	32	33,5
l	**30-aug-77**	32	33,5
l	**6-sep-77**	32	33,5
l	**13-sep-77**	32	33,5
l	**12-jul-77**	33	31
l	**19-jul-77**	33	31,5
l	**26-jul-77**	33	31,5
l	**2-aug-77**	33	31,5
l	**12-jul-77**	34	30,5
l	**19-jul-77**	34	32,5
l	**26-jul-77**	34	33,5
l	**2-aug-77**	34	33,5
l	**9-aug-77**	34	33,5
l	**16-aug-77**	34	33,5
l	**25-aug-77**	34	33,5
l	**30-aug-77**	34	33,5
l	**6-sep-77**	34	33,5
l	**12-jul-77**	35	31

I	19-jul-77	35	32
I	26-jul-77	35	32,5
I	2-aug-77	35	32,5
I	9-aug-77	35	32,5
I	16-aug-77	35	32,5
I	25-aug-77	35	32,5
I	30-aug-77	35	32,5
I	12-jul-77	36	32,5
I	19-jul-77	36	32,5
I	26-jul-77	36	33
I	2-aug-77	36	33,5
I	9-aug-77	36	33,5
I	16-aug-77	36	33,5
I	25-aug-77	36	33,5
I	30-aug-77	36	33,5
I	6-sep-77	36	33,5
I	12-jul-77	37	33
I	19-jul-77	37	33
I	26-jul-77	37	33,5
I	2-aug-77	37	34
I	9-aug-77	37	34
I	16-aug-77	37	34
I	25-aug-77	37	34
I	12-jul-77	38	32
I	19-jul-77	38	34,5
I	26-jul-77	38	35
I	2-aug-77	38	35
I	9-aug-77	38	35
I	16-aug-77	38	35
I	25-aug-77	38	35
I	30-aug-77	38	35
I	12-jul-77	39	33
I	19-jul-77	39	33,5
I	26-jul-77	39	33,5
I	2-aug-77	39	33,5
I	9-aug-77	39	33,5
I	16-aug-77	39	33,5
I	25-aug-77	39	33,5
I	30-aug-77	39	33,5
I	6-sep-77	39	33,5
I	19-jul-77	40	30,5
I	26-jul-77	40	32
I	2-aug-77	40	32
I	19-jul-77	41	31,5
I	26-jul-77	41	34
I	2-aug-77	41	34
I	9-aug-77	41	34
I	16-aug-77	41	34,5
I	25-aug-77	41	34,5
I	30-aug-77	41	35
I	6-sep-77	41	35
I	13-sep-77	41	35
I	20-sep-77	41	35
I	19-jul-77	42	33
I	26-jul-77	42	33,5
I	2-aug-77	42	33,5

I	9-aug-77	42	33,5
I	16-aug-77	42	33,5
I	25-aug-77	42	34
I	30-aug-77	42	34
I	6-sep-77	42	34
I	13-sep-77	42	34
I	19-jul-77	43	32,5
I	26-jul-77	43	33,5
I	2-aug-77	43	34
I	9-aug-77	43	34
I	16-aug-77	43	34
I	25-aug-77	43	34,5
I	30-aug-77	43	34,5
I	6-sep-77	43	34,5
I	19-jul-77	44	28,5
I	26-jul-77	44	28,5
I	2-aug-77	44	28,5
I	9-aug-77	44	29
I	16-aug-77	44	29
I	25-aug-77	44	29
I	19-jul-77	45	30
I	26-jul-77	45	30,5
I	2-aug-77	45	31
I	9-aug-77	45	31
I	16-aug-77	45	31
I	19-jul-77	46	30
I	26-jul-77	46	33
I	2-aug-77	46	33,5
I	9-aug-77	46	34
I	16-aug-77	46	34
I	25-aug-77	46	34
I	30-aug-77	46	34
I	6-sep-77	46	34,5
I	13-sep-77	46	34,5
I	20-sep-77	46	34,5
I	19-jul-77	47	30
I	26-jul-77	47	32,5
I	2-aug-77	47	34
I	9-aug-77	47	34
I	16-aug-77	47	34
I	25-aug-77	47	34
I	30-aug-77	47	34
I	6-sep-77	47	34
I	13-sep-77	47	34
I	19-jul-77	48	30,5
I	26-jul-77	48	30,5
I	2-aug-77	48	30,5
I	9-aug-77	48	30,5
I	19-jul-77	49	30
I	26-jul-77	49	31
I	2-aug-77	49	32,5
I	9-aug-77	49	32,5
I	16-aug-77	49	32,5
I	25-aug-77	49	32,5
I	30-aug-77	49	32,5
I	6-sep-77	49	32,5

I	**13-sep-77**	49	32,5
I	**19-jul-77**	50	31
I	**26-jul-77**	50	31
I	**2-aug-77**	50	32,5
I	**9-aug-77**	50	32,5
I	**16-aug-77**	50	32,5
I	**25-aug-77**	50	32,5
I	**30-aug-77**	50	32,5
I	**6-sep-77**	50	32,5
I	**13-sep-77**	50	32,5
I	**26-jul-77**	51	31,5
I	**2-aug-77**	51	34
I	**9-aug-77**	51	35
I	**16-aug-77**	51	35
I	**25-aug-77**	51	35,5
I	**30-aug-77**	51	35,5
I	**6-sep-77**	51	35,5
I	**13-sep-77**	51	35,5
I	**20-sep-77**	51	35,5
I	**27-sep-77**	51	35,5
I	**26-jul-77**	52	32
I	**2-aug-77**	52	32
I	**9-aug-77**	52	32
I	**16-aug-77**	52	32
I	**25-aug-77**	52	32
I	**30-aug-77**	52	32
I	**6-sep-77**	52	32
I	**26-jul-77**	53	33
I	**2-aug-77**	53	33
I	**9-aug-77**	53	33,5
I	**16-aug-77**	53	33,5
I	**25-aug-77**	53	33,5
I	**30-aug-77**	53	34
I	**6-sep-77**	53	34
I	**13-sep-77**	53	34
I	**20-sep-77**	53	34
I	**27-sep-77**	53	34
I	**26-jul-77**	54	31
I	**2-aug-77**	54	31
I	**9-aug-77**	54	31,5
I	**16-aug-77**	54	32
I	**25-aug-77**	54	32
I	**30-aug-77**	54	32
I	**2-aug-77**	55	28
I	**9-aug-77**	55	31
I	**16-aug-77**	55	31,5
I	**25-aug-77**	55	32
I	**30-aug-77**	55	32,5
I	**6-sep-77**	55	32,5
I	**13-sep-77**	55	32,5
I	**20-sep-77**	55	32,5
I	**27-sep-77**	55	32,5
I	**4-okt-77**	55	32,5
I	**2-aug-77**	56	29,5
I	**9-aug-77**	56	31,5
I	**16-aug-77**	56	32

l	**25-aug-77**	56	32,5
l	**30-aug-77**	56	32,5
l	**6-sep-77**	56	33,5
l	**13-sep-77**	56	33,5
l	**20-sep-77**	56	33,5
l	**27-sep-77**	56	33,5
l	**2-aug-77**	57	31
l	**9-aug-77**	57	31,5
l	**16-aug-77**	57	32
l	**25-aug-77**	57	32,5
l	**30-aug-77**	57	32,5
l	**6-sep-77**	57	32,5
l	**13-sep-77**	57	32,5
l	**20-sep-77**	57	32,5
l	**27-sep-77**	57	32,5
l	**2-aug-77**	58	34
l	**9-aug-77**	58	35
l	**16-aug-77**	58	35
l	**25-aug-77**	58	36
l	**30-aug-77**	58	36
l	**6-sep-77**	58	36
l	**13-sep-77**	58	36
l	**20-sep-77**	58	36
l	**27-sep-77**	58	36
l	**2-aug-77**	59	34
l	**9-aug-77**	59	35
l	**16-aug-77**	59	35
l	**25-aug-77**	59	35
l	**30-aug-77**	59	35
l	**6-sep-77**	59	35
l	**13-sep-77**	59	35
l	**20-sep-77**	59	35
l	**27-sep-77**	59	35
l	**16-aug-77**	60	34,5
l	**25-aug-77**	60	35,5
l	**30-aug-77**	60	35,5
l	**6-sep-77**	60	36
l	**13-sep-77**	60	36
l	**20-sep-77**	60	36
l	**27-sep-77**	60	36
l	**4-okt-77**	60	36
l	**12-okt-77**	60	36
l	**19-okt-77**	60	36
l	**24-okt-77**	60	36
l	**16-aug-77**	61	32,5
l	**25-aug-77**	61	32,5
l	**30-aug-77**	61	33
l	**6-sep-77**	61	33,5
l	**13-sep-77**	61	33,5
l	**20-sep-77**	61	33,5
l	**27-sep-77**	61	33,5
l	**4-okt-77**	61	33,5
l	**12-okt-77**	61	33,5
l	**19-okt-77**	61	33,5
l	**16-aug-77**	62	32,5
l	**25-aug-77**	62	32,5

l	**30-aug-77**	62	33
l	**6-sep-77**	62	33
l	**13-sep-77**	62	33,5
l	**20-sep-77**	62	33,5
l	**27-sep-77**	62	34
l	**4-okt-77**	62	34
l	**12-okt-77**	62	34
l	**19-okt-77**	62	34
l	**16-aug-77**	63	27,5
l	**25-aug-77**	63	30
l	**30-aug-77**	63	30
l	**6-sep-77**	63	31
l	**13-sep-77**	63	31
l	**20-sep-77**	63	31
l	**27-sep-77**	63	31
l	**4-okt-77**	63	31
l	**12-okt-77**	63	31
l	**16-aug-77**	64	32,5
l	**25-aug-77**	64	33,5
l	**30-aug-77**	64	34
l	**6-sep-77**	64	34
l	**13-sep-77**	64	34
l	**20-sep-77**	64	34
l	**16-aug-77**	65	29
l	**25-aug-77**	65	30
l	**30-aug-77**	65	30,5
l	**6-sep-77**	65	31
l	**13-sep-77**	65	31
l	**20-sep-77**	65	31
l	**27-sep-77**	65	31
l	**4-okt-77**	65	31
l	**12-okt-77**	65	31
l	**19-okt-77**	65	31
l	**24-okt-77**	65	31
l	**16-aug-77**	66	36
l	**25-aug-77**	66	38
l	**30-aug-77**	66	38
l	**6-sep-77**	66	38,5
l	**13-sep-77**	66	38,5
l	**20-sep-77**	66	39
l	**27-sep-77**	66	39
l	**4-okt-77**	66	39
l	**12-okt-77**	66	39
l	**19-okt-77**	66	39
l	**24-okt-77**	66	39
l	**1-nov-77**	66	39
l	**25-aug-77**	67	32
l	**30-aug-77**	67	33,5
l	**6-sep-77**	67	34
l	**13-sep-77**	67	34,5
l	**20-sep-77**	67	35
l	**27-sep-77**	67	35
l	**4-okt-77**	67	35
l	**12-okt-77**	67	35
l	**19-okt-77**	67	35
l	**24-okt-77**	67	35

l	1-nov-77	67	35
l	30-aug-77	68	34
l	6-sep-77	68	35
l	13-sep-77	68	35
l	20-sep-77	68	35,5
l	27-sep-77	68	35,5
l	4-okt-77	68	35,5
l	12-okt-77	68	35,5
l	19-okt-77	68	35,5
l	24-okt-77	68	35,5
l	1-nov-77	68	35,5
l	8-nov-77	68	35,5
l	15-nov-77	68	35,5
l	24-nov-77	68	35,5
l	6-sep-77	69	35,5
l	13-sep-77	69	35,5
l	20-sep-77	69	35,5
l	27-sep-77	69	35,5
l	4-okt-77	69	35,5
l	12-okt-77	69	35,5
l	19-okt-77	69	35,5
l	24-okt-77	69	35,5
l	1-nov-77	69	35,5
l	6-sep-77	70	32
l	13-sep-77	70	32,5
l	20-sep-77	70	32,5
l	27-sep-77	70	33
l	4-okt-77	70	33
l	12-okt-77	70	33
l	19-okt-77	70	33
l	24-okt-77	70	33
l	1-nov-77	70	33
l	8-nov-77	70	33
l	6-sep-77	71	33
l	13-sep-77	71	33
l	20-sep-77	71	34
l	27-sep-77	71	34
l	4-okt-77	71	34,5
l	12-okt-77	71	34,5
l	19-okt-77	71	34,5
l	24-okt-77	71	34,5
l	6-sep-77	72	31,5
l	13-sep-77	72	32,5
l	20-sep-77	72	32,5
l	27-sep-77	72	32,5
l	6-sep-77	73	28,5
l	13-sep-77	73	28,5
l	20-sep-77	73	29
l	27-sep-77	73	29
l	4-okt-77	73	29
l	12-okt-77	73	29
l	19-okt-77	73	29
l	24-okt-77	73	29
l	6-sep-77	74	32
l	13-sep-77	74	32,5
l	20-sep-77	74	32,5

I	**27-sep-77**	74	32,5
I	**4-okt-77**	74	32,5
I	**12-okt-77**	74	33
I	**19-okt-77**	74	33
I	**24-okt-77**	74	33
I	**1-nov-77**	74	33
I	**8-nov-77**	74	33
I	**15-nov-77**	74	33
I	**24-nov-77**	74	33
I	**13-sep-77**	75	30
I	**20-sep-77**	75	30
I	**27-sep-77**	75	30
I	**4-okt-77**	75	30,5
I	**12-okt-77**	75	30,5
I	**19-okt-77**	75	30,5
I	**24-okt-77**	75	30,5
I	**1-nov-77**	75	30,5
I	**8-nov-77**	75	30,5
I	**15-nov-77**	75	30,5
I	**24-nov-77**	75	30,5
I	**13-sep-77**	76	29
I	**20-sep-77**	76	29,5
I	**27-sep-77**	76	29,5
I	**13-sep-77**	77	30
I	**20-sep-77**	77	30,5
I	**27-sep-77**	77	30,5
I	**4-okt-77**	77	30,5
I	**12-okt-77**	77	30,5
I	**19-okt-77**	77	30,5
I	**24-okt-77**	77	30,5
I	**1-nov-77**	77	30,5
I	**8-nov-77**	77	30,5
I	**15-nov-77**	77	30,5
I	**24-nov-77**	77	30,5
J	**18-mei-77**	1	28
J	**23-mei-77**	1	28
J	**26-mei-77**	1	28
J	**1-jun-77**	1	29
J	**3-jun-77**	1	29
J	**6-jun-77**	1	29
J	**18-mei-77**	2	29
J	**23-mei-77**	2	31
J	**26-mei-77**	2	31,5
J	**1-jun-77**	2	32
J	**3-jun-77**	2	32
J	**6-jun-77**	2	32
J	**9-jun-77**	2	32
J	**13-jun-77**	2	32
J	**17-jun-77**	2	32
J	**20-jun-77**	2	32
J	**18-mei-77**	3	30
J	**23-mei-77**	3	30
J	**26-mei-77**	3	30
J	**1-jun-77**	3	30
J	**3-jun-77**	3	30
J	**6-jun-77**	3	30

J	**9-jun-77**	3	30
J	**13-jun-77**	3	30
J	**18-mei-77**	4	29
J	**23-mei-77**	4	30
J	**26-mei-77**	4	31
J	**1-jun-77**	4	31
J	**3-jun-77**	4	31
J	**6-jun-77**	4	31
J	**9-jun-77**	4	31
J	**13-jun-77**	4	31
J	**23-mei-77**	5	30
J	**26-mei-77**	5	31
J	**1-jun-77**	5	31
J	**3-jun-77**	5	31
J	**6-jun-77**	5	31
J	**9-jun-77**	5	31
J	**13-jun-77**	5	31
J	**26-mei-77**	6	30
J	**1-jun-77**	6	33
J	**3-jun-77**	6	33
J	**6-jun-77**	6	33
J	**9-jun-77**	6	33
J	**13-jun-77**	6	33
J	**1-jun-77**	7	33
J	**3-jun-77**	7	33
J	**6-jun-77**	7	34
J	**9-jun-77**	7	34
J	**13-jun-77**	7	34
J	**17-jun-77**	7	34
J	**20-jun-77**	7	34
J	**27-jun-77**	7	34
J	**1-jul-77**	7	34
J	**4-jul-77**	7	34
J	**8-jul-77**	7	34
J	**1-jun-77**	8	26,5
J	**3-jun-77**	8	26,5
J	**6-jun-77**	8	27
J	**9-jun-77**	8	27,5
J	**13-jun-77**	8	27,5
J	**3-jun-77**	9	34
J	**6-jun-77**	9	34
J	**9-jun-77**	9	35
J	**13-jun-77**	9	35
J	**17-jun-77**	9	35
J	**20-jun-77**	9	35,5
J	**27-jun-77**	9	35,5
J	**1-jul-77**	9	35,5
J	**4-jul-77**	9	35,5
J	**8-jul-77**	9	35,5
J	**6-jun-77**	10	23
J	**9-jun-77**	10	25
J	**13-jun-77**	10	25
J	**17-jun-77**	10	25
J	**20-jun-77**	10	25
J	**27-jun-77**	10	25
J	**1-jul-77**	10	25

J	**4-jul-77**	10	25
J	**8-jul-77**	10	25
J	**6-jun-77**	11	28
J	**9-jun-77**	11	29,5
J	**13-jun-77**	11	29,5
J	**17-jun-77**	11	29,5
J	**20-jun-77**	11	29,5
J	**27-jun-77**	11	30
J	**1-jul-77**	11	30
J	**4-jul-77**	11	30
J	**8-jul-77**	11	30
J	**6-jun-77**	12	25
J	**9-jun-77**	12	25
J	**13-jun-77**	12	25
J	**17-jun-77**	12	25
J	**20-jun-77**	12	25,5
J	**27-jun-77**	12	25,5
J	**1-jul-77**	12	25,5
J	**4-jul-77**	12	25,5
J	**8-jul-77**	12	25,5
J	**9-jun-77**	13	29
J	**13-jun-77**	13	29
J	**17-jun-77**	13	29
J	**20-jun-77**	13	29
J	**27-jun-77**	13	29
J	**1-jul-77**	13	29
J	**4-jul-77**	13	29
J	**8-jul-77**	13	29
J	**9-jun-77**	14	32
J	**13-jun-77**	14	34
J	**17-jun-77**	14	35
J	**20-jun-77**	14	35
J	**27-jun-77**	14	35
J	**1-jul-77**	14	35
J	**4-jul-77**	14	35
J	**8-jul-77**	14	35
J	**14-jul-77**	14	35
J	**17-jun-77**	15	36
J	**20-jun-77**	15	36
J	**27-jun-77**	15	37,5
J	**1-jul-77**	15	37,5
J	**4-jul-77**	15	37,5
J	**8-jul-77**	15	37,5
J	**14-jul-77**	15	37,5
J	**20-jul-77**	15	37,5
J	**27-jul-77**	15	37,5
J	**17-jun-77**	16	32
J	**20-jun-77**	16	32
J	**27-jun-77**	16	32
J	**1-jul-77**	16	32
J	**4-jul-77**	16	32
J	**8-jul-77**	16	32
J	**17-jun-77**	17	35
J	**20-jun-77**	17	35
J	**27-jun-77**	17	35,5
J	**1-jul-77**	17	36

J	**4-jul-77**	17	36
J	**8-jul-77**	17	36
J	**17-jun-77**	18	33
J	**20-jun-77**	18	33
J	**27-jun-77**	18	34
J	**1-jul-77**	18	34
J	**4-jul-77**	18	34
J	**8-jul-77**	18	34
J	**17-jun-77**	19	36,5
J	**20-jun-77**	19	37
J	**27-jun-77**	19	37
J	**1-jul-77**	19	37,5
J	**4-jul-77**	19	37,5
J	**8-jul-77**	19	37,5
J	**14-jul-77**	19	37,5
J	**20-jul-77**	19	37,5
J	**27-jul-77**	19	37,5
J	**17-jun-77**	20	27,5
J	**20-jun-77**	20	28,5
J	**27-jun-77**	20	30
J	**1-jul-77**	20	30
J	**4-jul-77**	20	30
J	**8-jul-77**	20	30
J	**14-jul-77**	20	30
J	**20-jul-77**	20	30
J	**27-jul-77**	20	30
J	**27-jun-77**	21	30
J	**1-jul-77**	21	31
J	**4-jul-77**	21	31,5
J	**8-jul-77**	21	32
J	**14-jul-77**	21	32
J	**20-jul-77**	21	32
J	**27-jul-77**	21	32
J	**3-aug-77**	21	32
J	**1-jul-77**	22	33
J	**4-jul-77**	22	34
J	**8-jul-77**	22	34
J	**14-jul-77**	22	35
J	**20-jul-77**	22	35
J	**27-jul-77**	22	35
J	**1-jul-77**	23	35
J	**4-jul-77**	23	35,5
J	**8-jul-77**	23	35,5
J	**14-jul-77**	23	36
J	**20-jul-77**	23	36
J	**27-jul-77**	23	36
J	**3-aug-77**	23	36
J	**1-jul-77**	24	33,5
J	**4-jul-77**	24	36
J	**8-jul-77**	24	37
J	**14-jul-77**	24	37
J	**20-jul-77**	24	37
J	**27-jul-77**	24	37
J	**3-aug-77**	24	37
J	**4-jul-77**	25	35
J	**8-jul-77**	25	35

J	14-jul-77	25	35
J	20-jul-77	25	35
J	27-jul-77	25	35
J	3-aug-77	25	35
J	4-jul-77	26	27
J	8-jul-77	26	29,5
J	14-jul-77	26	30,5
J	20-jul-77	26	30,5
J	27-jul-77	26	30,5
J	3-aug-77	26	30,5
J	8-jul-77	27	33
J	14-jul-77	27	34
J	20-jul-77	27	35,5
J	27-jul-77	27	35,5
J	3-aug-77	27	35,5
J	12-aug-77	27	35,5
J	19-aug-77	27	35,5
J	25-aug-77	27	35,5
J	8-jul-77	28	33,5
J	14-jul-77	28	34,5
J	20-jul-77	28	35,5
J	27-jul-77	28	36
J	3-aug-77	28	36,5
J	12-aug-77	28	36,5
J	19-aug-77	28	36,5
J	14-jul-77	29	30
J	20-jul-77	29	30,5
J	27-jul-77	29	30,5
J	3-aug-77	29	30,5
J	12-aug-77	29	30,5
J	19-aug-77	29	30,5
J	14-jul-77	30	31
J	20-jul-77	30	33,5
J	27-jul-77	30	34
J	3-aug-77	30	34
J	12-aug-77	30	34
J	19-aug-77	30	34
J	25-aug-77	30	34
J	14-jul-77	31	34
J	20-jul-77	31	35
J	27-jul-77	31	35
J	3-aug-77	31	35
J	12-aug-77	31	35
J	19-aug-77	31	35
J	25-aug-77	31	35
J	14-jul-77	32	35,5
J	20-jul-77	32	35,5
J	27-jul-77	32	36,5
J	3-aug-77	32	37
J	12-aug-77	32	37
J	19-aug-77	32	37
J	25-aug-77	32	37
J	31-aug-77	32	37
J	14-jul-77	33	33,5
J	20-jul-77	33	34
J	27-jul-77	33	34

J	**3-aug-77**	33	34
J	**12-aug-77**	33	34
J	**19-aug-77**	33	34
J	**20-jul-77**	34	28
J	**27-jul-77**	34	29
J	**3-aug-77**	34	29
J	**12-aug-77**	34	29
J	**19-aug-77**	34	29
J	**25-aug-77**	34	29
J	**31-aug-77**	34	29
J	**20-jul-77**	35	31
J	**27-jul-77**	35	32
J	**3-aug-77**	35	33
J	**12-aug-77**	35	33
J	**19-aug-77**	35	33
J	**25-aug-77**	35	33
J	**20-jul-77**	36	32,5
J	**27-jul-77**	36	33,5
J	**3-aug-77**	36	33,5
J	**12-aug-77**	36	33,5
J	**19-aug-77**	36	33,5
J	**25-aug-77**	36	33,5
J	**20-jul-77**	37	31
J	**27-jul-77**	37	33
J	**3-aug-77**	37	33,5
J	**12-aug-77**	37	34
J	**19-aug-77**	37	34
J	**25-aug-77**	37	34
J	**31-aug-77**	37	34
J	**27-jul-77**	38	33
J	**3-aug-77**	38	36,5
J	**12-aug-77**	38	36,5
J	**19-aug-77**	38	36,5
J	**25-aug-77**	38	36,5
J	**31-aug-77**	38	36,5
J	**7-sep-77**	38	36,5
J	**14-sep-77**	38	36,5
J	**27-jul-77**	39	29
J	**3-aug-77**	39	29,5
J	**12-aug-77**	39	29,5
J	**19-aug-77**	39	30
J	**25-aug-77**	39	30
J	**31-aug-77**	39	30
J	**27-jul-77**	40	32
J	**3-aug-77**	40	35,5
J	**12-aug-77**	40	37
J	**19-aug-77**	40	37
J	**25-aug-77**	40	37
J	**31-aug-77**	40	37
J	**7-sep-77**	40	37
J	**14-sep-77**	40	37
J	**21-sep-77**	40	37
J	**3-aug-77**	41	29
J	**12-aug-77**	41	30,5
J	**19-aug-77**	41	30,5
J	**25-aug-77**	41	30,5

J	31-aug-77	41	31
J	7-sep-77	41	31
J	14-sep-77	41	31
J	21-sep-77	41	31
J	28-sep-77	41	31
J	3-aug-77	42	31
J	12-aug-77	42	33,5
J	19-aug-77	42	33,5
J	25-aug-77	42	33,5
J	31-aug-77	42	34
J	7-sep-77	42	34
J	14-sep-77	42	34
J	21-sep-77	42	34
J	28-sep-77	42	34
J	12-aug-77	43	35
J	19-aug-77	43	37,5
J	25-aug-77	43	37,5
J	31-aug-77	43	37,5
J	7-sep-77	43	38,5
J	14-sep-77	43	38,5
J	21-sep-77	43	39
J	28-sep-77	43	39
J	3-okt-77	43	39
J	10-okt-77	43	39
J	18-okt-77	43	39
J	24-okt-77	43	39
J	12-aug-77	44	33,5
J	19-aug-77	44	35
J	25-aug-77	44	36
J	31-aug-77	44	36,5
J	7-sep-77	44	36,5
J	14-sep-77	44	36,5
J	21-sep-77	44	36,5
J	28-sep-77	44	36,5
J	3-okt-77	44	36,5
J	10-okt-77	44	36,5
J	18-okt-77	44	36,5
J	12-aug-77	45	37
J	19-aug-77	45	37,5
J	25-aug-77	45	37,5
J	31-aug-77	45	38
J	7-sep-77	45	38,5
J	14-sep-77	45	38,5
J	21-sep-77	45	38,5
J	28-sep-77	45	38,5
J	12-aug-77	46	32
J	19-aug-77	46	33,5
J	25-aug-77	46	33,5
J	31-aug-77	46	34
J	7-sep-77	46	34
J	14-sep-77	46	34
J	21-sep-77	46	34
J	28-sep-77	46	34
J	3-okt-77	46	34
J	10-okt-77	46	34
J	12-aug-77	47	14

J	**19-aug-77**	47	16,5
J	**25-aug-77**	47	16,5
J	**31-aug-77**	47	16,5
J	**7-sep-77**	47	17
J	**14-sep-77**	47	17
J	**21-sep-77**	47	17
J	**19-aug-77**	48	36,5
J	**25-aug-77**	48	38
J	**31-aug-77**	48	38
J	**7-sep-77**	48	38
J	**14-sep-77**	48	38
J	**21-sep-77**	48	38
J	**28-sep-77**	48	38
J	**3-okt-77**	48	38
J	**19-aug-77**	49	33,5
J	**25-aug-77**	49	33,5
J	**31-aug-77**	49	34
J	**7-sep-77**	49	34
J	**14-sep-77**	49	34
J	**21-sep-77**	49	34
J	**19-aug-77**	50	31,5
J	**25-aug-77**	50	31,5
J	**31-aug-77**	50	31,5
J	**7-sep-77**	50	31,5
J	**19-aug-77**	51	31,5
J	**25-aug-77**	51	31,5
J	**31-aug-77**	51	32
J	**7-sep-77**	51	32
J	**14-sep-77**	51	32
J	**21-sep-77**	51	32
J	**28-sep-77**	51	32
J	**3-okt-77**	51	32
J	**19-aug-77**	52	32
J	**25-aug-77**	52	33
J	**31-aug-77**	52	34
J	**7-sep-77**	52	34
J	**14-sep-77**	52	34
J	**21-sep-77**	52	34
J	**28-sep-77**	52	34
J	**3-okt-77**	52	34
J	**19-aug-77**	53	30
J	**25-aug-77**	53	30,5
J	**31-aug-77**	53	30,5
J	**7-sep-77**	53	30,5
J	**14-sep-77**	53	30,5
J	**21-sep-77**	53	30,5
J	**19-aug-77**	54	30
J	**25-aug-77**	54	33
J	**31-aug-77**	54	33,5
J	**7-sep-77**	54	33,5
J	**14-sep-77**	54	33,5
J	**21-sep-77**	54	33,5
J	**28-sep-77**	54	33,5
J	**25-aug-77**	55	28
J	**31-aug-77**	55	29
J	**7-sep-77**	55	29,5

J	**14-sep-77**	55	30
J	**21-sep-77**	55	30
J	**28-sep-77**	55	30
J	**25-aug-77**	56	29
J	**31-aug-77**	56	31
J	**7-sep-77**	56	31,5
J	**14-sep-77**	56	31,5
J	**21-sep-77**	56	31,5
J	**28-sep-77**	56	31,5
J	**3-okt-77**	56	31,5
J	**10-okt-77**	56	31,5
J	**25-aug-77**	57	33
J	**31-aug-77**	57	33
J	**7-sep-77**	57	33,5
J	**14-sep-77**	57	33,5
J	**21-sep-77**	57	34
J	**28-sep-77**	57	34
J	**3-okt-77**	57	34
J	**10-okt-77**	57	34
J	**18-okt-77**	57	34
J	**24-okt-77**	57	34
J	**1-nov-77**	57	34
J	**31-aug-77**	58	35
J	**7-sep-77**	58	35
J	**14-sep-77**	58	36
J	**21-sep-77**	58	36
J	**28-sep-77**	58	36
J	**3-okt-77**	58	36
J	**10-okt-77**	58	36
J	**18-okt-77**	58	36
J	**24-okt-77**	58	36
J	**1-nov-77**	58	36
J	**7-sep-77**	59	16,5
J	**14-sep-77**	59	17
J	**21-sep-77**	59	17
J	**28-sep-77**	59	17
K	**18-mei-77**	1	18
K	**23-mei-77**	1	20
K	**25-mei-77**	1	20
K	**26-mei-77**	1	20
K	**1-jun-77**	1	20
K	**6-jun-77**	1	20
K	**9-jun-77**	1	20
K	**13-jun-77**	1	20
K	**17-jun-77**	1	20
K	**20-jun-77**	1	20
K	**18-mei-77**	2	20
K	**23-mei-77**	2	21
K	**25-mei-77**	2	21
K	**26-mei-77**	2	21,5
K	**1-jun-77**	2	21,5
K	**6-jun-77**	2	22
K	**9-jun-77**	2	22
K	**13-jun-77**	2	22
K	**17-jun-77**	2	22
K	**23-mei-77**	3	21,5

K	25-mei-77	3	21,5
K	26-mei-77	3	21,5
K	23-mei-77	4	14
K	25-mei-77	4	16,5
K	26-mei-77	4	16,5
K	1-jun-77	4	17
K	6-jun-77	4	18
K	9-jun-77	4	18
K	13-jun-77	4	18
K	17-jun-77	4	18
K	20-jun-77	4	18
K	27-jun-77	4	18
K	1-jul-77	4	18
K	23-mei-77	5	21,5
K	25-mei-77	5	22
K	26-mei-77	5	23
K	1-jun-77	5	23
K	6-jun-77	5	24,5
K	9-jun-77	5	24,5
K	13-jun-77	5	24,5
K	17-jun-77	5	24,5
K	20-jun-77	5	24,5
K	27-jun-77	5	24,5
K	1-jul-77	5	24,5
K	23-mei-77	6	22
K	25-mei-77	6	22,5
K	26-mei-77	6	23
K	1-jun-77	6	24
K	6-jun-77	6	24
K	9-jun-77	6	25
K	13-jun-77	6	25
K	17-jun-77	6	25
K	20-jun-77	6	25
K	27-jun-77	6	25
K	1-jul-77	6	25
K	23-mei-77	7	21,5
K	25-mei-77	7	21,5
K	26-mei-77	7	22
K	1-jun-77	7	22
K	6-jun-77	7	23
K	9-jun-77	7	23
K	13-jun-77	7	23
K	17-jun-77	7	23,5
K	23-mei-77	8	20
K	25-mei-77	8	23
K	26-mei-77	8	23
K	25-mei-77	9	15,5
K	26-mei-77	9	15,5
K	26-mei-77	10	20
K	1-jun-77	10	20,5
K	6-jun-77	10	21
K	9-jun-77	10	21
K	13-jun-77	10	21
K	17-jun-77	10	21
K	20-jun-77	10	22
K	27-jun-77	10	22

K	1-jul-77	10	22
K	26-mei-77	11	22
K	1-jun-77	11	23,5
K	6-jun-77	11	24
K	9-jun-77	11	25
K	13-jun-77	11	25
K	17-jun-77	11	25
K	20-jun-77	11	25
K	27-jun-77	11	25
K	1-jul-77	11	26
K	6-jul-77	11	26
K	13-jul-77	11	26
K	26-mei-77	12	17
K	1-jun-77	12	17
K	6-jun-77	12	17
K	9-jun-77	12	19
K	13-jun-77	12	19
K	17-jun-77	12	19
K	26-mei-77	13	22
K	1-jun-77	13	23
K	6-jun-77	13	24
K	9-jun-77	13	24
K	13-jun-77	13	24
K	26-mei-77	14	22
K	1-jun-77	14	24,5
K	6-jun-77	14	26
K	9-jun-77	14	26
K	13-jun-77	14	26
K	17-jun-77	14	26,5
K	20-jun-77	14	26,5
K	27-jun-77	14	27
K	1-jul-77	14	27
K	6-jul-77	14	27
K	13-jul-77	14	27
K	26-mei-77	15	21,5
K	1-jun-77	15	24
K	6-jun-77	15	24
K	9-jun-77	15	25
K	13-jun-77	15	26
K	17-jun-77	15	26
K	20-jun-77	15	26
K	27-jun-77	15	26
K	1-jul-77	15	26
K	6-jul-77	15	26
K	13-jul-77	15	26
K	26-mei-77	16	23
K	1-jun-77	16	24,5
K	6-jun-77	16	24,5
K	9-jun-77	16	25
K	13-jun-77	16	25
K	17-jun-77	16	25
K	20-jun-77	16	26
K	27-jun-77	16	26
K	1-jul-77	16	26
K	6-jul-77	16	26
K	13-jul-77	16	26

K	**1-jun-77**	17	25
K	**6-jun-77**	17	26
K	**9-jun-77**	17	26,5
K	**13-jun-77**	17	27
K	**17-jun-77**	17	27,5
K	**20-jun-77**	17	27,5
K	**27-jun-77**	17	27,5
K	**1-jul-77**	17	28
K	**6-jul-77**	17	28
K	**13-jul-77**	17	28
K	**1-jun-77**	18	24
K	**6-jun-77**	18	26
K	**9-jun-77**	18	26,5
K	**13-jun-77**	18	27
K	**17-jun-77**	18	27,5
K	**20-jun-77**	18	28,5
K	**27-jun-77**	18	28,5
K	**1-jul-77**	18	28,5
K	**6-jul-77**	18	28,5
K	**13-jul-77**	18	28,5
K	**1-jun-77**	19	18
K	**6-jun-77**	19	19
K	**9-jun-77**	19	19
K	**13-jun-77**	19	19,5
K	**17-jun-77**	19	20
K	**20-jun-77**	19	20
K	**27-jun-77**	19	20
K	**1-jul-77**	19	20,5
K	**6-jul-77**	19	20,5
K	**1-jun-77**	20	22,5
K	**6-jun-77**	20	24
K	**9-jun-77**	20	25,5
K	**13-jun-77**	20	26
K	**17-jun-77**	20	27
K	**20-jun-77**	20	27
K	**27-jun-77**	20	27
K	**1-jul-77**	20	27
K	**6-jul-77**	20	27
K	**13-jul-77**	20	27
K	**6-jun-77**	21	19
K	**9-jun-77**	21	20,5
K	**13-jun-77**	21	21,5
K	**17-jun-77**	21	22
K	**20-jun-77**	21	22
K	**27-jun-77**	21	22
K	**1-jul-77**	21	22,5
K	**6-jul-77**	21	23
K	**13-jul-77**	21	23
K	**20-jul-77**	21	23
K	**6-jun-77**	22	25
K	**9-jun-77**	22	26,5
K	**13-jun-77**	22	27,5
K	**17-jun-77**	22	28,5
K	**20-jun-77**	22	28,5
K	**27-jun-77**	22	28,5
K	**1-jul-77**	22	29

K	6-jul-77	22	29
K	13-jul-77	22	29
K	20-jul-77	22	29
K	6-jun-77	23	10
K	9-jun-77	23	10
K	13-jun-77	23	10
K	17-jun-77	23	10,5
K	20-jun-77	23	10,5
K	6-jun-77	24	23,5
K	9-jun-77	24	26
K	13-jun-77	24	28
K	17-jun-77	24	28
K	20-jun-77	24	29
K	27-jun-77	24	30
K	1-jul-77	24	30
K	6-jul-77	24	30
K	13-jul-77	24	30
K	20-jul-77	24	30
K	13-jun-77	25	26
K	17-jun-77	25	29
K	20-jun-77	25	29
K	27-jun-77	25	30
K	1-jul-77	25	30
K	6-jul-77	25	30
K	13-jul-77	25	30
K	20-jul-77	25	30
K	27-jul-77	25	30
K	13-jun-77	26	26
K	17-jun-77	26	26
K	13-jun-77	27	26,5
K	17-jun-77	27	28,5
K	20-jun-77	27	29
K	27-jun-77	27	29,5
K	1-jul-77	27	30,5
K	6-jul-77	27	30,5
K	13-jul-77	27	30,5
K	20-jul-77	27	30,5
K	27-jul-77	27	30,5
K	13-jun-77	28	9,5
K	17-jun-77	28	10,5
K	20-jun-77	28	10,5
K	27-jun-77	28	11
K	1-jul-77	28	11
K	17-jun-77	29	27
K	20-jun-77	29	27,5
K	27-jun-77	29	28,5
K	1-jul-77	29	29
K	6-jul-77	29	29
K	13-jul-77	29	29
K	20-jul-77	29	29
K	27-jul-77	29	29
K	3-aug-77	29	29
K	17-jun-77	30	27
K	20-jun-77	30	28
K	27-jun-77	30	30
K	1-jul-77	30	30,5

K	**6-jul-77**	30	31
K	**13-jul-77**	30	31
K	**20-jul-77**	30	31
K	**27-jul-77**	30	31
K	**3-aug-77**	30	31
K	**11-aug-77**	30	31
K	**17-jun-77**	31	11
K	**20-jun-77**	31	11
K	**27-jun-77**	31	11
K	**17-jun-77**	32	7,8
K	**20-jun-77**	32	8
K	**27-jun-77**	32	8
K	**1-jul-77**	32	8
K	**6-jul-77**	32	8
K	**17-jun-77**	33	27,5
K	**20-jun-77**	33	29
K	**27-jun-77**	33	30
K	**1-jul-77**	33	30,5
K	**6-jul-77**	33	31
K	**13-jul-77**	33	31
K	**20-jul-77**	33	31
K	**17-jun-77**	34	20,5
K	**20-jun-77**	34	22
K	**27-jun-77**	34	23
K	**1-jul-77**	34	23,5
K	**6-jul-77**	34	23,5
K	**13-jul-77**	34	23,5
K	**20-jul-77**	34	23,5
K	**20-jun-77**	35	12
K	**27-jun-77**	35	13,5
K	**1-jul-77**	35	14
K	**6-jul-77**	35	14
K	**13-jul-77**	35	14
K	**27-jun-77**	36	20,5
K	**1-jul-77**	36	23,5
K	**6-jul-77**	36	25
K	**13-jul-77**	36	25
K	**20-jul-77**	36	25
K	**27-jul-77**	36	25
K	**27-jun-77**	37	28
K	**1-jul-77**	37	29,5
K	**6-jul-77**	37	30
K	**13-jul-77**	37	30
K	**20-jul-77**	37	30
K	**27-jul-77**	37	30
K	**3-aug-77**	37	30
K	**27-jun-77**	38	28,5
K	**1-jul-77**	38	30
K	**6-jul-77**	38	30,5
K	**13-jul-77**	38	31
K	**20-jul-77**	38	31
K	**27-jul-77**	38	31
K	**3-aug-77**	38	31
K	**11-aug-77**	38	31
K	**1-jul-77**	39	27,5
K	**6-jul-77**	39	29

K	13-jul-77	39	30
K	20-jul-77	39	30,5
K	27-jul-77	39	31
K	3-aug-77	39	31
K	11-aug-77	39	31
K	19-aug-77	39	31,5
K	26-aug-77	39	31,5
K	31-aug-77	39	31,5
K	1-jul-77	40	19
K	6-jul-77	40	19
K	1-jul-77	41	8
K	6-jul-77	41	8,5
K	13-jul-77	41	8,5
K	1-jul-77	42	31,5
K	6-jul-77	42	31,5
K	13-jul-77	42	31,5
K	20-jul-77	42	31,5
K	27-jul-77	42	31,5
K	3-aug-77	42	31,5
K	11-aug-77	42	31,5
K	19-aug-77	42	31,5
K	1-jul-77	43	29
K	6-jul-77	43	30
K	13-jul-77	43	30,5
K	20-jul-77	43	31
K	27-jul-77	43	31
K	3-aug-77	43	31
K	11-aug-77	43	31
K	19-aug-77	43	31
K	1-jul-77	44	23
K	6-jul-77	44	24
K	13-jul-77	44	24
K	20-jul-77	44	24
K	6-jul-77	46	29,5
K	13-jul-77	46	29,5
K	20-jul-77	46	29,5
K	6-jul-77	47	30
K	13-jul-77	47	30,5
K	20-jul-77	47	31
K	27-jul-77	47	32
K	3-aug-77	47	32
K	11-aug-77	47	33
K	19-aug-77	47	33
K	26-aug-77	47	33
K	31-aug-77	47	33
K	7-sep-77	47	33
K	14-sep-77	47	33
K	6-jul-77	48	22,5
K	13-jul-77	48	25
K	20-jul-77	48	25,5
K	27-jul-77	48	25,5
K	3-aug-77	48	26
K	11-aug-77	48	26
K	6-jul-77	49	20
K	13-jul-77	49	20,5
K	20-jul-77	49	20,5

K	**27-jul-77**	49	20,5
K	**3-aug-77**	49	21
K	**11-aug-77**	49	21
K	**19-aug-77**	49	21
K	**26-aug-77**	49	21
K	**6-jul-77**	50	21
K	**13-jul-77**	50	21,5
K	**20-jul-77**	50	22
K	**27-jul-77**	50	22
K	**3-aug-77**	50	22
K	**11-aug-77**	50	22
K	**6-jul-77**	51	24
K	**13-jul-77**	51	25,5
K	**20-jul-77**	51	26
K	**27-jul-77**	51	26
K	**3-aug-77**	51	26
K	**11-aug-77**	51	26
K	**13-jul-77**	52	26
K	**20-jul-77**	52	27
K	**27-jul-77**	52	28
K	**3-aug-77**	52	28
K	**11-aug-77**	52	29
K	**19-aug-77**	52	29
K	**26-aug-77**	52	29
K	**31-aug-77**	52	29
K	**7-sep-77**	52	29
K	**14-sep-77**	52	29
K	**13-jul-77**	53	20,5
K	**20-jul-77**	53	21
K	**27-jul-77**	53	21
K	**3-aug-77**	53	21,5
K	**11-aug-77**	53	21,5
K	**13-jul-77**	54	23,5
K	**20-jul-77**	54	26
K	**27-jul-77**	54	27
K	**3-aug-77**	54	27,5
K	**11-aug-77**	54	27,5
K	**19-aug-77**	54	28
K	**26-aug-77**	54	28
K	**31-aug-77**	54	28
K	**7-sep-77**	54	28
K	**14-sep-77**	54	28
K	**13-jul-77**	55	20
K	**20-jul-77**	55	22
K	**27-jul-77**	55	23
K	**3-aug-77**	55	23,5
K	**11-aug-77**	55	24
K	**19-aug-77**	55	24
K	**26-aug-77**	55	24
K	**13-jul-77**	56	29
K	**20-jul-77**	56	30
K	**27-jul-77**	56	30,5
K	**3-aug-77**	56	31
K	**11-aug-77**	56	31,5
K	**19-aug-77**	56	31,5
K	**26-aug-77**	56	32

K	**31-aug-77**	56	32
K	**7-sep-77**	56	32
K	**14-sep-77**	56	32
K	**13-jul-77**	57	28
K	**20-jul-77**	57	30
K	**27-jul-77**	57	30
K	**3-aug-77**	57	30,5
K	**11-aug-77**	57	31
K	**19-aug-77**	57	31
K	**26-aug-77**	57	31
K	**31-aug-77**	57	31
K	**13-jul-77**	58	7
K	**20-jul-77**	58	8
K	**27-jul-77**	58	8
K	**3-aug-77**	58	8
K	**11-aug-77**	58	8
K	**13-jul-77**	59	5,5
K	**20-jul-77**	59	6
K	**27-jul-77**	59	6
K	**13-jul-77**	60	12
K	**20-jul-77**	60	14
K	**27-jul-77**	60	14
K	**3-aug-77**	60	14
K	**13-jul-77**	61	24
K	**20-jul-77**	61	24,5
K	**27-jul-77**	61	25
K	**3-aug-77**	61	25
K	**11-aug-77**	61	25,5
K	**19-aug-77**	61	25,5
K	**26-aug-77**	61	25,5
K	**13-jul-77**	62	28
K	**20-jul-77**	62	29,5
K	**27-jul-77**	62	30
K	**3-aug-77**	62	30,5
K	**11-aug-77**	62	30,5
K	**19-aug-77**	62	30,5
K	**26-aug-77**	62	30,5
K	**13-jul-77**	63	33
K	**20-jul-77**	63	33,5
K	**27-jul-77**	63	34
K	**3-aug-77**	63	34
K	**11-aug-77**	63	34
K	**13-jul-77**	64	22,5
K	**20-jul-77**	64	25,5
K	**27-jul-77**	64	26
K	**3-aug-77**	64	26
K	**11-aug-77**	64	27
K	**19-aug-77**	64	27
K	**26-aug-77**	64	27
K	**31-aug-77**	64	27
K	**13-jul-77**	65	25
K	**20-jul-77**	65	25,5
K	**27-jul-77**	65	25,5
K	**3-aug-77**	65	26
K	**11-aug-77**	65	26
K	**19-aug-77**	65	26

K	13-jul-77	66	28
K	20-jul-77	66	28,5
K	27-jul-77	66	28,5
K	3-aug-77	66	28,5
K	11-aug-77	66	28,5
K	19-aug-77	66	28,5
K	13-jul-77	67	14
K	20-jul-77	67	14,5
K	27-jul-77	67	14,5
K	3-aug-77	67	14,5
K	11-aug-77	67	14,5
K	13-jul-77	68	7,5
K	20-jul-77	68	9
K	27-jul-77	68	9,5
K	3-aug-77	68	9,5
K	13-jul-77	69	9
K	20-jul-77	69	9,5
K	27-jul-77	69	9,5
K	3-aug-77	69	9,5
K	13-jul-77	70	25
K	20-jul-77	70	27,5
K	27-jul-77	70	28
K	3-aug-77	70	28
K	11-aug-77	70	28,5
K	19-aug-77	70	28,5
K	26-aug-77	70	28,5
K	31-aug-77	70	28,5
K	20-jul-77	71	28
K	27-jul-77	71	28,5
K	3-aug-77	71	29
K	11-aug-77	71	30
K	19-aug-77	71	30
K	26-aug-77	71	30
K	31-aug-77	71	30
K	7-sep-77	71	30
K	20-jul-77	72	25
K	27-jul-77	72	26,5
K	3-aug-77	72	28
K	11-aug-77	72	29
K	19-aug-77	72	29
K	26-aug-77	72	29
K	31-aug-77	72	29
K	7-sep-77	72	29
K	14-sep-77	72	29
K	20-jul-77	73	24
K	27-jul-77	73	26,5
K	3-aug-77	73	28,5
K	11-aug-77	73	29
K	19-aug-77	73	30
K	26-aug-77	73	30
K	31-aug-77	73	30
K	7-sep-77	73	30
K	14-sep-77	73	30
K	20-jul-77	74	8
K	27-jul-77	74	8
K	3-aug-77	74	8

K	**20-jul-77**	75	5,5
K	**27-jul-77**	75	5,5
K	**3-aug-77**	75	5,5
K	**20-jul-77**	76	10
K	**27-jul-77**	76	10,5
K	**3-aug-77**	76	10,5
K	**11-aug-77**	76	10,5
K	**19-aug-77**	76	11
K	**26-aug-77**	76	11
K	**31-aug-77**	76	11
K	**20-jul-77**	77	30,5
K	**27-jul-77**	77	31
K	**3-aug-77**	77	32
K	**11-aug-77**	77	32
K	**19-aug-77**	77	32
K	**26-aug-77**	77	32,5
K	**31-aug-77**	77	32,5
K	**7-sep-77**	77	32,5
K	**20-jul-77**	78	23,5
K	**27-jul-77**	78	26
K	**3-aug-77**	78	27
K	**11-aug-77**	78	27
K	**19-aug-77**	78	27,5
K	**26-aug-77**	78	28
K	**31-aug-77**	78	28
K	**20-jul-77**	79	22,5
K	**27-jul-77**	79	24,5
K	**3-aug-77**	79	25
K	**11-aug-77**	79	26
K	**19-aug-77**	79	26
K	**26-aug-77**	79	26
K	**31-aug-77**	79	26
K	**27-jul-77**	80	9
K	**3-aug-77**	80	9,5
K	**11-aug-77**	80	10
K	**19-aug-77**	80	10
K	**27-jul-77**	81	28,5
K	**3-aug-77**	81	30,5
K	**11-aug-77**	81	31
K	**19-aug-77**	81	31,5
K	**26-aug-77**	81	32
K	**31-aug-77**	81	32
K	**7-sep-77**	81	32
K	**14-sep-77**	81	32
K	**21-sep-77**	81	32
K	**28-sep-77**	81	32
K	**27-jul-77**	82	20
K	**3-aug-77**	82	22
K	**11-aug-77**	82	24
K	**19-aug-77**	82	24
K	**26-aug-77**	82	25
K	**31-aug-77**	82	25
K	**7-sep-77**	82	25
K	**14-sep-77**	82	25
K	**21-sep-77**	82	25
K	**27-jul-77**	83	29

K	3-aug-77	83	30,5
K	11-aug-77	83	30,5
K	19-aug-77	83	31
K	26-aug-77	83	31
K	31-aug-77	83	31,5
K	7-sep-77	83	31,5
K	3-aug-77	84	26,5
K	11-aug-77	84	29
K	19-aug-77	84	29
K	26-aug-77	84	30
K	31-aug-77	84	30
K	7-sep-77	84	30
K	14-sep-77	84	30,5
K	21-sep-77	84	30,5
K	28-sep-77	84	30,5
K	3-okt-77	84	30,5
K	10-okt-77	84	30,5
K	19-okt-77	84	30,5
K	3-aug-77	85	26,5
K	11-aug-77	85	28,5
K	19-aug-77	85	29
K	26-aug-77	85	29
K	31-aug-77	85	30
K	7-sep-77	85	30,5
K	14-sep-77	85	30,5
K	21-sep-77	85	30,5
K	28-sep-77	85	30,5
K	3-okt-77	85	30,5
K	10-okt-77	85	30,5
K	3-aug-77	86	26
K	11-aug-77	86	27,5
K	19-aug-77	86	27,5
K	26-aug-77	86	27,5
K	31-aug-77	86	28
K	7-sep-77	86	28
K	14-sep-77	86	28
K	21-sep-77	86	28
K	3-aug-77	87	13,5
K	11-aug-77	87	14
K	19-aug-77	87	14
K	3-aug-77	88	29
K	11-aug-77	88	31
K	19-aug-77	88	31,5
K	26-aug-77	88	32
K	31-aug-77	88	32,5
K	7-sep-77	88	32,5
K	14-sep-77	88	32,5
K	3-aug-77	89	26
K	11-aug-77	89	28,5
K	19-aug-77	89	28,5
K	26-aug-77	89	29
K	31-aug-77	89	29
K	7-sep-77	89	29
K	14-sep-77	89	29
K	21-sep-77	89	29
K	28-sep-77	89	29

K	**11-aug-77**	90	26
K	**19-aug-77**	90	27
K	**26-aug-77**	90	27
K	**31-aug-77**	90	27,5
K	**7-sep-77**	90	27,5
K	**14-sep-77**	90	27,5
K	**11-aug-77**	91	22,5
K	**19-aug-77**	91	24
K	**26-aug-77**	91	24,5
K	**31-aug-77**	91	25
K	**7-sep-77**	91	25
K	**14-sep-77**	91	25
K	**21-sep-77**	91	25
K	**28-sep-77**	91	25
K	**3-okt-77**	91	25
K	**10-okt-77**	91	25
K	**19-okt-77**	91	25
K	**11-aug-77**	92	26
K	**19-aug-77**	92	28,5
K	**26-aug-77**	92	29
K	**31-aug-77**	92	29
K	**7-sep-77**	92	30
K	**14-sep-77**	92	30,5
K	**21-sep-77**	92	30,5
K	**28-sep-77**	92	30,5
K	**3-okt-77**	92	30,5
K	**10-okt-77**	92	30,5
K	**19-okt-77**	92	30,5
K	**11-aug-77**	93	21
K	**19-aug-77**	93	21
K	**26-aug-77**	93	22
K	**31-aug-77**	93	22
K	**7-sep-77**	93	22
K	**14-sep-77**	93	22
K	**21-sep-77**	93	22
K	**28-sep-77**	93	22
K	**11-aug-77**	94	25
K	**19-aug-77**	94	25,5
K	**26-aug-77**	94	26
K	**31-aug-77**	94	26,5
K	**7-sep-77**	94	26,5
K	**14-sep-77**	94	26,5
K	**11-aug-77**	95	25
K	**19-aug-77**	95	26
K	**26-aug-77**	95	27
K	**31-aug-77**	95	27
K	**7-sep-77**	95	27,5
K	**14-sep-77**	95	27,5
K	**21-sep-77**	95	27,5
K	**28-sep-77**	95	27,5
K	**3-okt-77**	95	27,5
K	**10-okt-77**	95	27,5
K	**11-aug-77**	96	26
K	**19-aug-77**	96	28,5
K	**26-aug-77**	96	29
K	**31-aug-77**	96	29

K	7-sep-77	96	29,5
K	14-sep-77	96	29,5
K	21-sep-77	96	29,5
K	28-sep-77	96	30
K	3-okt-77	96	30
K	10-okt-77	96	30
K	19-okt-77	96	30
K	24-okt-77	96	30
K	11-aug-77	97	27,5
K	19-aug-77	97	28
K	26-aug-77	97	28
K	31-aug-77	97	29
K	7-sep-77	97	29
K	14-sep-77	97	29
K	21-sep-77	97	30
K	28-sep-77	97	30
K	3-okt-77	97	30
K	10-okt-77	97	30
K	19-okt-77	97	30
K	19-aug-77	98	26
K	26-aug-77	98	26
K	31-aug-77	98	26
K	7-sep-77	98	26
K	14-sep-77	98	26
K	21-sep-77	98	26
K	28-sep-77	98	26
K	3-okt-77	98	26
K	10-okt-77	98	26
K	19-okt-77	98	26
K	24-okt-77	98	26
K	19-aug-77	99	26
K	26-aug-77	99	27
K	31-aug-77	99	27
K	7-sep-77	99	28
K	14-sep-77	99	28
K	21-sep-77	99	28
K	28-sep-77	99	28
K	3-okt-77	99	28
K	10-okt-77	99	28
K	19-okt-77	99	28
K	24-okt-77	99	28
K	1-nov-77	99	28
K	19-aug-77	100	24
K	26-aug-77	100	25
K	31-aug-77	100	25
K	19-aug-77	101	21
K	26-aug-77	101	22,5
K	31-aug-77	101	22,5
K	7-sep-77	101	22,5
K	14-sep-77	101	23
K	21-sep-77	101	23,5
K	28-sep-77	101	23,5
K	3-okt-77	101	23,5
K	19-aug-77	102	21
K	26-aug-77	102	24
K	31-aug-77	102	24,5

K	7-sep-77	102	25
K	14-sep-77	102	25
K	21-sep-77	102	25
K	28-sep-77	102	25
K	3-okt-77	102	25
K	10-okt-77	102	25
K	26-aug-77	103	15,5
K	31-aug-77	103	17,5
K	7-sep-77	103	18,5
K	14-sep-77	103	19
K	21-sep-77	103	19,5
K	28-sep-77	103	19,5
K	3-okt-77	103	19,5
K	10-okt-77	103	19,5
K	19-okt-77	103	19,5
K	24-okt-77	103	19,5
K	26-aug-77	104	23,5
K	31-aug-77	104	25,5
K	7-sep-77	104	26,5
K	14-sep-77	104	27
K	21-sep-77	104	27,5
K	28-sep-77	104	27,5
K	3-okt-77	104	27,5
K	10-okt-77	104	27,5
K	19-okt-77	104	27,5
K	26-aug-77	105	23
K	31-aug-77	105	25
K	7-sep-77	105	25
K	14-sep-77	105	25,5
K	21-sep-77	105	25,5
K	28-sep-77	105	26
K	3-okt-77	105	26
K	10-okt-77	105	26
K	19-okt-77	105	26
K	24-okt-77	105	26
K	1-nov-77	105	26
K	31-aug-77	106	27,5
K	7-sep-77	106	29
K	14-sep-77	106	29,5
K	21-sep-77	106	29,5
K	28-sep-77	106	29,5
K	3-okt-77	106	29,5
K	10-okt-77	106	29,5
K	19-okt-77	106	29,5
K	24-okt-77	106	29,5
K	1-nov-77	106	29,5
K	31-aug-77	107	25
K	7-sep-77	107	26
K	14-sep-77	107	26,5
K	21-sep-77	107	26,5
K	28-sep-77	107	26,5
K	3-okt-77	107	26,5
K	10-okt-77	107	26,5
K	19-okt-77	107	26,5
K	7-sep-77	108	19,5
K	14-sep-77	108	21

K	21-sep-77	108	21
K	28-sep-77	108	21
K	3-okt-77	108	21
K	10-okt-77	108	21
K	19-okt-77	108	21
K	24-okt-77	108	21
K	1-nov-77	108	21
K	7-sep-77	109	21,5
K	14-sep-77	109	22,5
K	21-sep-77	109	23,5
K	28-sep-77	109	23,5
K	3-okt-77	109	23,5
K	10-okt-77	109	23,5
K	19-okt-77	109	23,5
K	24-okt-77	109	23,5
K	1-nov-77	109	23,5
K	6-nov-77	109	23,5
L	14-jun-77	1	15,5
L	21-jun-77	1	17,5
L	28-jun-77	1	17,5
L	5-jul-77	1	17,5
L	12-jul-77	1	17,5
L	19-jul-77	1	17,5
L	26-jul-77	1	17,5
L	2-aug-77	1	17,5
L	9-aug-77	1	17,5
L	14-jun-77	2	16
L	21-jun-77	2	17,5
L	28-jun-77	2	17,5
L	5-jul-77	2	17,5
L	12-jul-77	2	17,5
L	19-jul-77	2	17,5
L	26-jul-77	2	17,5
L	2-aug-77	2	17,5
L	14-jun-77	3	14,5
L	21-jun-77	3	16
L	28-jun-77	3	16
L	5-jul-77	3	16,5
L	12-jul-77	3	16,5
L	19-jul-77	3	16,5
L	14-jun-77	4	15,5
L	21-jun-77	4	16
L	28-jun-77	4	16
L	5-jul-77	4	16
L	12-jul-77	4	16
L	19-jul-77	4	16
L	26-jul-77	4	16
L	2-aug-77	4	16
L	14-jun-77	5	15,5
L	21-jun-77	5	18
L	28-jun-77	5	19
L	5-jul-77	5	19
L	12-jul-77	5	19
L	19-jul-77	5	19
L	26-jul-77	5	19
L	2-aug-77	5	19

L	9-aug-77	5	19
L	14-jun-77	6	16,5
L	21-jun-77	6	17
L	28-jun-77	6	17
L	5-jul-77	6	17
L	12-jul-77	6	17,5
L	19-jul-77	6	17,5
L	26-jul-77	6	17,5
L	2-aug-77	6	17,5
L	14-jun-77	7	16
L	21-jun-77	7	17
L	28-jun-77	7	17
L	5-jul-77	7	17
L	12-jul-77	7	17
L	19-jul-77	7	17,5
L	26-jul-77	7	17,5
L	2-aug-77	7	17,5
L	14-jun-77	8	13,5
L	21-jun-77	8	14
L	28-jun-77	8	14
L	5-jul-77	8	14
L	12-jul-77	8	14
L	21-jun-77	9	15,5
L	28-jun-77	9	16
L	5-jul-77	9	16,5
L	12-jul-77	9	16,5
L	19-jul-77	9	16,5
L	26-jul-77	9	16,5
L	2-aug-77	9	16,5
L	21-jun-77	10	16
L	28-jun-77	10	17
L	5-jul-77	10	18
L	12-jul-77	10	18
L	19-jul-77	10	18
L	26-jul-77	10	18
L	2-aug-77	10	18
L	9-aug-77	10	18
L	21-jun-77	11	17
L	28-jun-77	11	18
L	5-jul-77	11	19
L	12-jul-77	11	19
L	19-jul-77	11	19
L	26-jul-77	11	19
L	2-aug-77	11	19
L	9-aug-77	11	19
L	21-jun-77	12	16
L	28-jun-77	12	17,8
L	5-jul-77	12	17,8
L	28-jun-77	13	16,5
L	5-jul-77	13	16,5
L	28-jun-77	14	18
L	5-jul-77	14	19
L	12-jul-77	14	19
L	28-jun-77	15	7,5
L	5-jul-77	15	7,5
L	12-jul-77	15	7,5

L	28-jun-77	16	17,5
L	5-jul-77	16	19
L	12-jul-77	16	19
L	28-jun-77	17	16,5
L	5-jul-77	17	17
L	12-jul-77	17	17
L	5-jul-77	18	18,5
L	12-jul-77	18	19,5
L	19-jul-77	18	20
L	26-jul-77	18	20
L	2-aug-77	18	20
L	9-aug-77	18	20,5
L	16-aug-77	18	20,5
L	25-aug-77	18	21
L	30-aug-77	18	21
L	6-sep-77	18	21
L	13-sep-77	18	21
L	5-jul-77	19	21
L	12-jul-77	19	22
L	19-jul-77	19	22
L	26-jul-77	19	22
L	2-aug-77	19	22
L	9-aug-77	19	22
L	16-aug-77	19	22
L	25-aug-77	19	22,5
L	30-aug-77	19	22,5
L	6-sep-77	19	22,5
L	13-sep-77	19	22,5
L	5-jul-77	20	19
L	12-jul-77	20	19,5
L	19-jul-77	20	19,5
L	26-jul-77	20	19,5
L	2-aug-77	20	19,5
L	9-aug-77	20	20
L	16-aug-77	20	20
L	25-aug-77	20	20
L	5-jul-77	21	20,5
L	12-jul-77	21	21
L	19-jul-77	21	21
L	5-jul-77	22	19,5
L	12-jul-77	22	20
L	19-jul-77	22	20,5
L	26-jul-77	22	20,5
L	2-aug-77	22	20,5
L	9-aug-77	22	20,5
L	5-jul-77	23	19
L	12-jul-77	23	20
L	19-jul-77	23	20
L	26-jul-77	23	20
L	2-aug-77	23	20
L	9-aug-77	23	20,5
L	16-aug-77	23	20,5
L	25-aug-77	23	20,5
L	12-jul-77	24	20,5
L	19-jul-77	24	21,5
L	26-jul-77	24	22

L	2-aug-77	24	22
L	9-aug-77	24	22
L	16-aug-77	24	22
L	25-aug-77	24	22,5
L	30-aug-77	24	23
L	6-sep-77	24	23
L	13-sep-77	24	23
L	20-sep-77	24	23
L	27-sep-77	24	23
L	4-okt-77	24	23
L	12-okt-77	24	23
L	12-jul-77	25	21,5
L	19-jul-77	25	22,5
L	26-jul-77	25	22,5
L	2-aug-77	25	23
L	9-aug-77	25	23
L	16-aug-77	25	23
L	25-aug-77	25	23
L	30-aug-77	25	23
L	12-jul-77	26	21
L	19-jul-77	26	21,5
L	26-jul-77	26	22
L	2-aug-77	26	22
L	9-aug-77	26	22
L	16-aug-77	26	22
L	25-aug-77	26	22
L	30-aug-77	26	22
L	6-sep-77	26	22
L	12-jul-77	27	18
L	19-jul-77	27	18
L	26-jul-77	27	18
L	2-aug-77	27	18
L	9-aug-77	27	18
L	16-aug-77	27	18
L	25-aug-77	27	18
L	12-jul-77	28	20
L	19-jul-77	28	21
L	26-jul-77	28	21,5
L	2-aug-77	28	22
L	9-aug-77	28	22
L	16-aug-77	28	22
L	25-aug-77	28	22
L	30-aug-77	28	22,5
L	6-sep-77	28	22,5
L	13-sep-77	28	22,5
L	20-sep-77	28	22,5
L	12-jul-77	29	19,5
L	19-jul-77	29	19,5
L	26-jul-77	29	19,5
L	2-aug-77	29	19,5
L	9-aug-77	29	19,5
L	12-jul-77	30	18
L	19-jul-77	30	20
L	26-jul-77	30	20,5
L	2-aug-77	30	20,5
L	9-aug-77	30	21

L	16-aug-77	30	21
L	25-aug-77	30	21,5
L	30-aug-77	30	21,5
L	6-sep-77	30	22
L	13-sep-77	30	22
L	20-sep-77	30	22
L	19-jul-77	31	19,5
L	26-jul-77	31	19,5
L	2-aug-77	31	20
L	9-aug-77	31	20
L	16-aug-77	31	20,5
L	25-aug-77	31	20,5
L	30-aug-77	31	21
L	6-sep-77	31	21
L	13-sep-77	31	21
L	20-sep-77	31	21
L	19-jul-77	32	18,5
L	26-jul-77	32	19
L	2-aug-77	32	19
L	9-aug-77	32	20
L	16-aug-77	32	20
L	25-aug-77	32	20
L	30-aug-77	32	20
L	19-jul-77	33	20,5
L	26-jul-77	33	21
L	2-aug-77	33	21
L	9-aug-77	33	21,5
L	16-aug-77	33	21,5
L	25-aug-77	33	22
L	30-aug-77	33	22,5
L	6-sep-77	33	22,5
L	13-sep-77	33	23
L	20-sep-77	33	23
L	27-sep-77	33	23
L	4-okt-77	33	23
L	19-jul-77	34	20,5
L	26-jul-77	34	21
L	2-aug-77	34	21
L	9-aug-77	34	21,5
L	16-aug-77	34	21,5
L	25-aug-77	34	22
L	30-aug-77	34	22
L	6-sep-77	34	22
L	26-jul-77	35	20
L	2-aug-77	35	22
L	9-aug-77	35	22,5
L	16-aug-77	35	22,5
L	25-aug-77	35	22,5
L	30-aug-77	35	23
L	6-sep-77	35	23
L	13-sep-77	35	23,5
L	20-sep-77	35	23,5
L	27-sep-77	35	23,5
L	4-okt-77	35	23,5
L	26-jul-77	36	20,5
L	2-aug-77	36	21

L	9-aug-77	36	21
L	16-aug-77	36	21
L	25-aug-77	36	21
L	30-aug-77	36	21
L	6-sep-77	36	21
L	2-aug-77	37	18,5
L	9-aug-77	37	20,5
L	16-aug-77	37	20,5
L	25-aug-77	37	21
L	30-aug-77	37	21,5
L	6-sep-77	37	21,5
L	13-sep-77	37	22
L	20-sep-77	37	22
L	27-sep-77	37	22
L	4-okt-77	37	22
L	2-aug-77	38	19,5
L	9-aug-77	38	20
L	16-aug-77	38	20
L	25-aug-77	38	20
L	30-aug-77	38	20
L	6-sep-77	38	20
L	2-aug-77	39	18
L	9-aug-77	39	19
L	16-aug-77	39	19
L	25-aug-77	39	19
L	30-aug-77	39	19,5
L	6-sep-77	39	20
L	13-sep-77	39	20
L	20-sep-77	39	20
L	27-sep-77	39	20
L	4-okt-77	39	20
L	12-okt-77	39	20
L	2-aug-77	40	18
L	9-aug-77	40	18,5
L	16-aug-77	40	19
L	25-aug-77	40	19,5
L	30-aug-77	40	19,5
L	6-sep-77	40	19,5
L	9-aug-77	41	16
L	16-aug-77	41	19
L	25-aug-77	41	19,5
L	30-aug-77	41	19,5
L	6-sep-77	41	19,5
L	13-sep-77	41	20
L	20-sep-77	41	20
L	27-sep-77	41	20
L	4-okt-77	41	20
L	12-okt-77	41	20
L	19-okt-77	41	20
L	16-aug-77	42	14
L	25-aug-77	42	14
L	30-aug-77	42	14
L	6-sep-77	42	14,5
L	13-sep-77	42	14,5
L	20-sep-77	42	14,5
L	27-sep-77	42	14,5

L	**16-aug-77**	43	13,5
L	**25-aug-77**	43	13,5
L	**30-aug-77**	43	13,5
L	**6-sep-77**	43	13,5
L	**13-sep-77**	43	13,5
L	**20-sep-77**	43	13,5
L	**27-sep-77**	43	13,5

candida
