# Supplementary material for: Plant traits and environment: floating leaf blade production and turnover of waterlilies
Source: PeerJ. 2017 Apr 27;5:e3212. doi: 10.7717/peerj.3212 (PMC5410161; doi:10.7717/peerj.3212)
Supplement: Data S2 [file peerj-05-3212-s002.pdf]

Raw data 1988: Week, Plot, Leaf number, Leaf length

Plot: M = VG, Nymphaea alba; N = VG, Nuphar lutea

Week	Plot	Leaf number	Leaf length (cm)
0	M	1	13,2
1	M	1	14,3
2	M	1	15,6
3	M	1	16,2
4	M	1	16,3
0	M	2	13,5
1	M	2	15,9
2	M	2	16,5
3	M	2	16,4
4	M	2	16,2
0	M	3	14,7
1	M	3	15,2
2	M	3	17,7
3	M	3	17,8
4	M	3	17,8
1	M	4	14,7
2	M	4	15,9
3	M	4	16,1
4	M	4	16,3
1	M	5	13,9
2	M	5	16,4
3	M	5	16,7
1	M	6	14,6
2	M	6	15,9
3	M	6	16
4	M	6	16,2
1	M	7	14,3
2	M	7	15,7
3	M	7	15,8
4	M	7	16,2
1	M	8	15,1
2	M	8	15,4
3	M	8	15,5
4	M	8	15,7
2	M	9	15,9
3	M	9	16,9
4	M	9	17,6
5	M	9	17,6
6	M	9	17,6
2	M	10	15,8
3	M	10	15,7
4	M	10	16
5	M	10	16
2	M	11	16,4
3	M	11	16,4
4	M	11	16,4
5	M	11	16,4
2	M	12	16,3
3	M	12	16,8
4	M	12	16,8
2	M	13	14
3	M	13	16

**4**	M	13	16,1
**5**	M	13	16,4
**6**	M	13	16,5
**3**	M	14	14,9
**4**	M	14	16,3
**5**	M	14	16,7
**6**	M	14	17
**7**	M	14	17
**8**	M	14	17,8
**3**	M	15	18,6
**4**	M	15	19,5
**5**	M	15	20,1
**3**	M	16	19,3
**4**	M	16	20,3
**5**	M	16	20,7
**6**	M	16	21
**7**	M	16	20,8
**3**	M	17	19,3
**4**	M	17	20,3
**5**	M	17	20,3
**6**	M	17	20,7
**7**	M	17	21
**8**	M	17	21
**3**	M	18	18,6
**4**	M	18	18,6
**5**	M	18	19,1
**6**	M	18	19,3
**4**	M	19	17,3
**5**	M	19	18,6
**6**	M	19	18,6
**7**	M	19	18,8
**8**	M	19	18,7
**9**	M	19	19
**4**	M	20	17,5
**5**	M	20	19,9
**6**	M	20	20,3
**7**	M	20	20,4
**8**	M	20	20,4
**9**	M	20	20,6
**4**	M	21	19,8
**5**	M	21	20,5
**6**	M	21	20,5
**7**	M	21	20,4
**8**	M	21	21,7
**9**	M	21	21,3
**4**	M	22	19
**6**	M	22	19
**7**	M	22	20,9
**8**	M	22	20,3
**9**	M	22	20,5
**4**	M	23	16,5
**5**	M	23	17,7
**6**	M	23	18,3
**7**	M	23	18,3
**8**	M	23	18,6
**9**	M	23	18,3

5	M	28	12,3
6	M	28	12,3
7	M	28	12,6
8	M	28	12,9
5	M	29	7,3
6	M	29	8,2
7	M	29	8,2
5	M	30	19,8
6	M	30	20,2
7	M	30	20,9
8	M	30	21,1
9	M	30	21,2
10	M	30	21,2
11	M	30	21,6
5	M	31	19,8
6	M	31	20,4
7	M	31	20,5
8	M	31	20,6
9	M	31	20,8
5	M	32	17,3
6	M	32	19,5
7	M	32	20,5
8	M	32	20,7
9	M	32	20,8
10	M	32	21
11	M	32	21
12	M	32	21,3
5	M	33	18,6
6	M	33	18,2
7	M	33	18,3
8	M	33	18,9
9	M	33	18,6
5	M	34	19
6	M	34	20,7
7	M	34	19,5
8	M	34	19,5
9	M	34	19,7
10	M	34	20,2
11	M	34	20,7
6	M	35	19,8
7	M	35	21,5
8	M	35	22,3
9	M	35	22,3
10	M	35	22,3
11	M	35	23
6	M	36	20,1
7	M	36	21,3
8	M	36	21,6
9	M	36	21,6
10	M	36	22,5
6	M	37	16,6
7	M	37	16,6
8	M	37	16,3
6	M	38	19
7	M	38	20,1
8	M	38	20,4

9	M	38	20,5
10	M	38	20,9
11	M	38	20,9
12	M	38	21
7	M	40	18,4
8	M	40	20,2
9	M	40	20,2
10	M	40	21,6
11	M	40	21,4
12	M	40	21,2
13	M	40	21,2
14	M	40	21,1
7	M	41	21,5
8	M	41	22,6
7	M	42	18,7
8	M	42	19,1
9	M	42	19,7
10	M	42	20,1
11	M	42	20,1
12	M	42	20,2
13	M	42	20,2
14	M	42	20,2
7	M	43	20,5
8	M	43	20,2
9	M	43	21,3
10	M	43	21,6
11	M	43	21,7
12	M	43	21,7
7	M	44	21,9
8	M	44	23
9	M	44	23,2
10	M	44	23,2
11	M	44	23,9
12	M	44	23,2
7	M	45	19,9
8	M	45	20,6
9	M	45	20,6
10	M	45	21,2
11	M	45	20,6
7	M	46	19,3
8	M	46	20
9	M	46	20,5
10	M	46	21,8
11	M	46	22,1
12	M	46	21,8
13	M	46	21,8
14	M	46	21,8
8	M	47	21,6
9	M	47	22,7
10	M	47	24,2
11	M	47	24,5
12	M	47	24,7
13	M	47	24,7
14	M	47	24,8
15	M	47	24,8
16	M	47	25,2

**17**	M	47	25,2
**18**	M	47	25,1
**19**	M	47	25,1
**8**	M	48	20,7
**9**	M	48	22,3
**10**	M	48	22,4
**11**	M	48	22,3
**12**	M	48	22,5
**13**	M	48	22,5
**14**	M	48	22,7
**15**	M	48	22,7
**16**	M	48	22,7
**8**	M	50	7,5
**9**	M	50	8,3
**10**	M	50	8,1
**8**	M	51	16,7
**9**	M	51	19,3
**10**	M	51	20,1
**11**	M	51	20,6
**12**	M	51	20,7
**13**	M	51	20,7
**14**	M	51	21,3
**15**	M	51	21,3
**16**	M	51	20,6
**8**	M	52	17,7
**9**	M	52	20,3
**10**	M	52	20,2
**11**	M	52	20,2
**12**	M	52	20,2
**13**	M	52	20,2
**14**	M	52	20,5
**15**	M	52	20,5
**16**	M	52	20,1
**17**	M	52	20,1
**18**	M	52	20
**8**	M	53	21,7
**9**	M	53	21,7
**10**	M	53	22,6
**11**	M	53	22,6
**8**	M	54	20,9
**9**	M	54	21,3
**10**	M	54	21,6
**11**	M	54	22,1
**12**	M	54	22,1
**13**	M	54	22,1
**14**	M	54	22,9
**8**	M	55	14,8
**9**	M	55	15,5
**10**	M	55	15,9
**11**	M	55	16,3
**12**	M	55	16,4
**13**	M	55	16,4
**14**	M	55	16,4
**15**	M	55	16,4
**16**	M	55	16,2
**8**	M	56	20,7

**9**	M	56	22,2
**10**	M	56	23
**11**	M	56	23,1
**12**	M	56	23,1
**13**	M	56	23,1
**14**	M	56	23,2
**15**	M	56	23
**9**	M	57	23,2
**10**	M	57	23,8
**11**	M	57	23,8
**12**	M	57	24
**13**	M	57	24
**14**	M	57	23,8
**15**	M	57	23,8
**16**	M	57	23,9
**9**	M	58	18
**10**	M	58	20,7
**11**	M	58	20,7
**12**	M	58	21
**13**	M	58	21
**14**	M	58	21
**15**	M	58	21
**16**	M	58	21,1
**17**	M	58	21,1
**9**	M	59	18,6
**10**	M	59	21,6
**11**	M	59	21,6
**12**	M	59	21,9
**13**	M	59	21,9
**14**	M	59	22,5
**15**	M	59	22,4
**16**	M	59	22,4
**17**	M	59	22,4
**18**	M	59	22,5
**19**	M	59	22,5
**20**	M	59	22,2
**9**	M	60	20,9
**10**	M	60	22,1
**11**	M	60	20,6
**12**	M	60	22,6
**13**	M	60	22,4
**14**	M	60	22,4
**15**	M	60	22,4
**16**	M	60	22,4
**10**	M	61	21,3
**11**	M	61	20,6
**12**	M	61	20,5
**13**	M	61	20,7
**14**	M	61	20,7
**15**	M	61	20,6
**16**	M	61	20,6
**10**	M	62	20,9
**10**	M	63	19,8
**11**	M	63	19,8
**12**	M	63	20
**13**	M	63	20

14	M	63	20,2
15	M	63	20,2
16	M	63	20,2
17	M	63	20,2
18	M	63	20,4
19	M	63	20,4
10	M	64	13,6
11	M	64	14,5
12	M	64	14,8
13	M	64	14,8
14	M	64	14,9
10	M	65	20,5
11	M	65	21,3
12	M	65	21,6
13	M	65	21,6
14	M	65	21,8
15	M	65	21,8
16	M	65	21,8
17	M	65	21,8
18	M	65	22,2
19	M	65	22,2
20	M	65	22,1
11	M	66	19,2
12	M	66	19,9
13	M	66	19,9
14	M	66	20
15	M	66	20
16	M	66	20,3
17	M	66	20,3
18	M	66	20,2
19	M	66	20,2
11	M	67	21,1
12	M	67	21,9
13	M	67	21,9
14	M	67	22
15	M	67	22
16	M	67	22,7
17	M	67	22,7
18	M	67	22,8
19	M	67	22,8
10	M	67	22,7
12	M	68	20
13	M	68	20,4
14	M	68	20,3
15	M	68	20,5
16	M	68	20,5
17	M	68	20,5
18	M	68	20,6
19	M	68	20,6
20	M	68	20,6
12	M	69	17,2
13	M	69	17,2
14	M	69	17,9
15	M	69	17,9
16	M	69	18,5
17	M	69	19,1

**18**	M	69	19,1
**19**	M	69	19,1
**20**	M	69	19,1
**21**	M	69	19,1
**22**	M	69	19,1
**12**	M	70	14,9
**13**	M	70	14,9
**14**	M	70	15,9
**15**	M	70	15,9
**16**	M	70	16,2
**17**	M	70	16,2
**18**	M	70	16,6
**19**	M	70	16,6
**12**	M	71	18
**13**	M	71	19,5
**14**	M	71	19,5
**15**	M	71	20,5
**16**	M	71	20,3
**17**	M	71	20,3
**18**	M	71	20,4
**19**	M	71	20,3
**20**	M	71	20,4
**21**	M	71	20,3
**22**	M	71	20,6
**12**	M	72	16,1
**13**	M	72	17,1
**14**	M	72	17,1
**15**	M	72	17,5
**16**	M	72	17,4
**17**	M	72	17,4
**18**	M	72	18,2
**19**	M	72	18,2
**20**	M	72	18,3
**21**	M	72	18,3
**22**	M	72	18,2
**12**	M	73	15,8
**13**	M	73	16,8
**14**	M	73	17,3
**15**	M	73	18
**16**	M	73	18
**17**	M	73	18
**18**	M	73	17,9
**19**	M	73	17,9
**20**	M	73	17,6
**13**	M	74	13
**14**	M	74	14
**15**	M	74	13,9
**16**	M	74	14,2
**14**	M	75	15,2
**15**	M	75	15,2
**16**	M	75	15,7
**17**	M	75	15,7
**18**	M	75	16
**19**	M	75	16
**20**	M	75	16,3
**21**	M	75	16,3

**22**	M	75	16,3
**14**	M	76	16,9
**15**	M	76	17,7
**16**	M	76	17,7
**17**	M	76	17,7
**18**	M	76	18
**19**	M	76	18
**20**	M	76	18
**21**	M	76	18
**22**	M	76	18
**23**	M	76	18
**14**	M	77	15
**15**	M	77	16
**16**	M	77	16
**17**	M	77	16,5
**18**	M	77	16,5
**19**	M	77	16,5
**20**	M	77	16,5
**14**	M	78	19,3
**15**	M	78	20,1
**16**	M	78	20
**17**	M	78	20
**18**	M	78	20
**14**	M	79	14,2
**15**	M	79	14,2
**16**	M	79	15
**17**	M	79	15
**18**	M	79	15,9
**19**	M	79	15,9
**20**	M	79	15,8
**21**	M	79	15,8
**22**	M	79	15,8
**23**	M	79	15,8
**14**	M	80	11,2
**15**	M	80	11,2
**16**	M	80	13
**17**	M	80	13
**18**	M	80	13,5
**19**	M	80	13,5
**20**	M	80	13,6
**21**	M	80	13,6
**22**	M	80	13,6
**23**	M	80	13,6
**14**	M	81	16,9
**15**	M	81	16,9
**16**	M	81	17,3
**17**	M	81	17,5
**18**	M	81	18,4
**19**	M	81	18,4
**20**	M	81	19
**21**	M	81	19
**22**	M	81	19
**23**	M	81	19
**16**	M	82	16,8
**17**	M	82	16,8
**18**	M	82	16,8

**19**	M	82	16,8
**20**	M	82	16,8
**21**	M	82	16,8
**22**	M	82	17
**23**	M	82	17
**24**	M	82	17
**16**	M	83	14,9
**17**	M	83	14,9
**18**	M	83	14,9
**19**	M	83	14,9
**20**	M	83	16,1
**21**	M	83	16,1
**22**	M	83	16,1
**16**	M	84	16,9
**17**	M	84	13,8
**18**	M	84	13,8
**19**	M	84	13,8
**20**	M	84	13,8
**21**	M	84	13,8
**22**	M	84	13,8
**23**	M	84	13,5
**24**	M	84	13,5
**18**	M	85	14,7
**19**	M	85	14,7
**18**	M	86	13,9
**19**	M	86	13,9
**20**	M	86	13,9
**21**	M	86	13,9
**22**	M	86	13,9
**23**	M	86	13,9
**24**	M	86	13,9
**25**	M	86	13,9
**0**	N	1	23,8
**1**	N	1	24
**2**	N	1	24
**3**	N	1	24,2
**4**	N	1	24,4
**5**	N	1	25,7
**6**	N	1	25,8
**2**	N	2	28,4
**3**	N	2	28,7
**4**	N	2	29,3
**5**	N	2	29,3
**6**	N	2	30
**7**	N	2	29,5
**3**	N	3	26,1
**4**	N	3	27,3
**5**	N	3	27,9
**6**	N	3	27,8
**7**	N	3	28,2
**3**	N	4	17,9
**4**	N	4	19
**5**	N	4	19,4
**6**	N	4	19,4
**7**	N	4	19,1
**4**	N	5	24,8

**5**	N	5	26,4
**6**	N	5	27,1
**7**	N	5	27,1
**8**	N	5	27,1
**9**	N	5	27,2
**10**	N	5	27,2
**6**	N	6	31,3
**7**	N	6	31,7
**8**	N	6	31,7
**9**	N	6	31,7
**7**	N	7	26,2
**8**	N	7	26,2
**9**	N	7	27,5
**10**	N	7	27,3
**11**	N	7	28,5
**12**	N	7	28,5
**7**	N	8	17,4
**8**	N	8	17,4
**9**	N	8	18,1
**10**	N	8	18,3
**9**	N	9	26,3
**10**	N	9	26,5
**11**	N	9	26,8
**12**	N	9	27,3
**13**	N	9	27,3
**14**	N	9	27,3
**15**	N	9	27,3
**9**	N	11	32,3
**10**	N	11	32,6
**11**	N	11	32,7
**12**	N	11	33
**13**	N	11	33
**14**	N	11	33,2
**10**	N	12	24,3
**11**	N	12	24,3
**12**	N	12	24,3
**13**	N	12	24,3
**14**	N	12	24,3
**15**	N	12	24,3
**10**	N	13	26,9
**11**	N	13	27,9
**12**	N	13	28,2
**13**	N	13	28,2
**14**	N	13	28,2
**15**	N	13	28,2
**10**	N	14	14,5
**11**	N	14	16
**12**	N	14	16,3
**13**	N	14	16,3
**14**	N	14	16,3
**15**	N	14	16,3
**11**	N	15	22,6
**12**	N	15	23,1
**13**	N	15	23,3
**14**	N	15	23,2
**15**	N	15	23,1

**12**	N	16	23,9
**13**	N	16	23,9
**14**	N	16	24,9
**15**	N	16	25,2
**16**	N	16	27,6
**17**	N	16	27,4
**18**	N	16	27,8
**19**	N	16	27,6
**20**	N	16	27,6
**21**	N	16	27,6
**22**	N	16	27,6
**23**	N	16	27,6
**24**	N	16	27,3
**12**	N	17	16,3
**13**	N	17	16,3
**14**	N	17	16,5
**15**	N	17	16,4
**12**	N	18	23,4
**13**	N	18	23,4
**14**	N	18	23,6
**15**	N	18	23,6
**16**	N	18	23,6
**17**	N	18	23,7
**18**	N	18	24
**19**	N	18	23,8
**20**	N	18	23,8
**21**	N	18	23,8
**22**	N	18	23,8
**23**	N	18	23,8
**24**	N	18	23,6
**15**	N	19	15,2
**16**	N	19	15,2
**17**	N	19	15,2
**18**	N	19	15,2
**19**	N	19	15
**20**	N	19	15,3
**21**	N	19	15,3
**15**	N	20	26,4
**16**	N	20	26,5
**17**	N	20	26,7
**18**	N	20	26,7
**19**	N	20	26,7
**20**	N	20	26,4
**21**	N	20	26,4
**22**	N	20	26,6
**23**	N	20	26,7
**24**	N	20	26,7
**25**	N	20	26,7
**16**	N	21	24,3
**17**	N	21	24,7
**18**	N	21	24,7
**19**	N	21	24,7
**20**	N	21	24,6
**21**	N	21	24,6
**22**	N	21	24,6
**23**	N	21	24,6

**24**	N	21	24,6
**25**	N	21	24,3
**16**	N	22	21,6
**17**	N	22	21,6
**18**	N	22	21,9
**19**	N	22	21,8
**20**	N	22	21,8
**21**	N	22	21,8
**18**	N	23	16,3
**19**	N	23	16,5
**21**	N	23	16,5
**22**	N	23	16,7
**23**	N	23	16,7
**24**	N	23	16,6
